# Improving biomedical entity linking with generative relevance feedback

**DOI:** 10.1093/bioinformatics/btag011

**Published:** 2026-01-14

**Authors:** Darya Shlyk, Lawrence Hunter

**Affiliations:** Department of Computer Science, Università degli Studi di Milano, Milan 20133, Italy; Department of Pediatrics, The University of Chicago, Chicago IL 60637, United States

## Abstract

**Motivation:**

Biomedical Entity Linking (BEL) maps mentions in biomedical text to standardized identifiers, enabling structured data integration and downstream knowledge discovery. However, current BEL systems remain fundamentally constrained by the recall of the initial candidate pool, where suboptimal retrieval limits the overall effectiveness of the normalization pipeline.

**Results:**

We present the first systematic evaluation of *Generative Relevance Feedback* (GRF) for enhancing candidate retrieval in state-of-the-art BEL systems. GRF leverages large language models (LLMs) to enrich the expressiveness of the mention in a zero-shot fashion. We assess GRF’s impact under two scenarios—direct linking prediction and candidate generation in cascading normalization pipelines—and analyze its sensitivity to different LLMs, feedback types, and integration strategies. Experiments across eight corpora and four biomedical knowledge bases demonstrate that integrating GRF significantly improves both accuracy and recall, thereby increasing the upper bound on normalization performance. Our findings highlight GRF as an efficient, model-agnostic solution and underscore its potential as a key component for advancing BEL.

**Availability and implementation:**

The code to reproduce our experiments can be found at: https://doi.org/10.5281/zenodo.17853541.

## 1 Introduction

Biomedical Entity Linking (the task is also known as Concept Normalization, Entity Disambiguation, or Grounding. We will use “entity linking”, and “normalizing” interchangeably throughout the text) (BEL) refers to the task of mapping biomedical entity mentions from unstructured text to unique concept identifiers within an expert-curated knowledge base (KB) or vocabulary (for simplicity, we will use “knowledge base (KB)” to refer to any type of normalization target, including controlled vocabularies, thesauri, and ontologies) (Abdulnazar et al. 2024). As a critical component of biomedical text mining pipelines, BEL enables the structured representation of textual information, thereby supporting a wide range of downstream applications such as knowledge base construction (Schäfer et al. 2024), information retrieval (Sutton et al. 2020), and literature-based discovery (Pu et al. 2025).

Research in BEL has traditionally focused on developing specialized models to learn dense representations of biomedical entities (Gillick et al. 2019 inter alia; Liu et al. 2020; Sung et al. 2020; Agarwal et al. 2022; Garda and Leser 2024). These representations can be used directly to make linking predictions. For instance, name-based BEL systems, such as BioSyn (Sung et al. 2020), treat the mention span as a query to find a matching concept name in the target KB based on semantic similarity between entity mention and concept embeddings. More frequently, these embeddings are employed within cascading two-stage entity linking pipelines, where candidate retrieval is followed by a re-ranking step. In this later stage, a more expressive model is typically used to refine the initial retrieval results and find the best match for a given mention from a narrow set of candidates (French and Mcinnes 2023). Recent advances in BEL increasingly leverage large language models (LLMs) as second-stage re-rankers through Retrieval-Augmented Generation (RAG) (Lewis et al. 2020). In this setting, LLMs contribute contextual understanding capabilities that enable a more nuanced assessment of the alignment between mention contexts and retrieved candidates, facilitating accurate normalization without additional task-specific training (Berkowitz et al. 2025). However, the effectiveness of these methods is fundamentally constrained by the *bounded recall problem*: if correct concepts are missed during candidate retrieval, they cannot be recovered downstream, regardless of how effective the re-ranking method is (MacAvaney et al. 2022).

To mitigate this issue, query expansion techniques, such as Pseudo-Relevance Feedback (PRF), propose to modify the query representation in an attempt to improve recall at the candidate retrieval stage (Li et al. 2021a, 2021b). Traditional PRF methods use information from top-ranked candidates, treating them as pseudo-relevant documents to construct an updated query for a second retrieval pass. More recently, Generative Relevance Feedback (GRF) has emerged as a novel strategy leveraging LLM’s text generation capabilities for zero-shot query expansion (Mackie et al. 2023a, 2023b). Unlike PRF, GRF does not require an additional retrieval pass. Instead, it prompts a general-purpose LLM to generate contextually relevant feedback on-the-fly, that serves to build a stronger query for more effective retrieval. Recent efforts applying GRF to enhance mention representation in BEL include zero-shot definition generation (Shlyk et al. 2024), alternative phrasing (Dobbins 2024), mention simplification (Borchert et al. 2024), and acronym resolution (Abdulnazar et al. 2024). While these studies demonstrate promising results, the potential of the GRF mechanism in BEL remains underexplored and lacks systematic evaluation.

To address this gap, we present a systematic investigation of the GRF potential for enhancing state-of-the-art BEL systems and evaluate its impact under two settings. **Direct linking prediction:** We examine whether GRF can improve retrieval effectiveness for direct linking, with emphasis on *accuracy*, i.e. the ability of the system to return the correct concept as top-1 result. **Candidate retrieval for downstream normalization:** We investigate the potential of GRF to increase the upper bound on normalization performance in cascading entity-linking pipelines. Here, we focus on *recall* of the candidate pool at small cutoff values (*k*) and analyze how recall changes as a result of GRF. Because our primary goal is to identify effective strategies that leverage GRF within BEL systems for enhanced retrieval effectiveness, we focus on the candidate retrieval stage, deferring the exploration of re-ranking methods to future work. Specifically, this study addresses the following research questions:

- **RQ1:** How to effectively leverage GRF for enhancing candidate retrieval effectiveness in BEL? (Section 4.1)
- **RQ2:** To what extent does GRF-enhanced retrieval improve normalization performance in state-of-the-art BEL systems? (Section 4.1)
- **RQ3:** What is the benefit of GRF-informed retrieval for down-stream normalization in cascading BEL pipelines? (Section 4.3)

## 2 Methods

In BEL, we assume that given a document corpus, D, all mentions of biomedical entities have already been identified (Most text mining pipelines precede entity linking with a Named Entity Recognition (NER) step, which detects entity spans in the text prior to linking them to a knowledge base). Let M denote the set of mention spans in D requiring normalization. Then, given a KB containing a set of curated biomedical concepts C, the task is to predict the correct concept c∈C for each mention span m∈M.

### 2.1 Index construction with KB homonymy

In the KB, each concept c∈C is associated with a unique identifier and a set of surface forms, or aliases, that serve as alternative concept names. For example, “atelosteogenesis, type 1,” “AO1,” “giant cell chondrodysplasia,” and “spondylohumerofemoral hypoplasia” all denote the same concept MESH: C535396, with “atelosteogenesis, type 1” designated as the preferred concept name. Name-based linking methods construct an index from unique names in the KB and then match a mention to the concept identifier with the most similar name in the index. However, homonymous names, that is, aliases that are shared by multiple distinct concepts, represent a practical issue for this linking approach, since they lead to name collisions during index construction. For instance, in the CTD Disease KB, the name “conorenal syndrome” serves as alias for two different conditions—“short rib–polydactyly syndrome” (MESH: D012779) and “Mainzer–Saldino disease” (MESH: C535463). These names should be disambiguated before the retrieval takes place so that each surface form in the index maps unambiguously to a single concept identifier in the KB and name-based linking can function correctly. We use the homonym disambiguation approach introduced in BELHD (Garda and Leser, 2024), and preprocess each KB at the index construction stage, replacing all duplicate names with disambiguated variants. For example, the homonym “conorenal syndrome” is changed to “conorenal syndrome (short rib–polydactyly syndrome)” and “conorenal syndrome (Mainzer–Saldino disease)” for MESH: D012779 and MESH: C535463, respectively. We then build the index I using aliases from the disambiguated KB. Additional details are provided in Supplementary Data Section 3, available as supplementary data at *Bioinformatics* online.

### 2.2 Candidate retrieval

For each disambiguated name in I, a vector representation is precomputed, using a pre-trained embedding model and stored in a vector database for efficient retrieval. The mention span is encoded using the same embedding model and used as a query, $Q_{0}$, to retrieve a ranked list of top-*k* candidate names from the index, $R^{k}=[s_{1},\ldots ,s_{k}]$. Cosine similarity is used as the distance metric to identify the *k* closest candidate vectors to $Q_{0}$. It is important to note that, although retrieval is performed over surface forms, evaluation is conducted at the concept level. Retrieved names are mapped to their corresponding concept identifiers, and only the highest-scoring surface form for each concept in $R^{k}$ is retained to produce the final candidate ranking.

### 2.3 Generative relevance feedback

The objective of GRF is to improve the effectiveness of candidate retrieval—specifically, to achieve high recall at small values of *k*—by enhancing the expressiveness of mention-based queries. The method modifies the query through targeted text-generation, encouraging the search system to promote relevant matches in the retrieved candidate pool. Prior to retrieval, the LLM is prompted to generate mention-specific feedback F, that is, contextually relevant textual content designed to semantically enrich the mention query $Q_{0}$. The candidate search then uses a new query, $Q_{\text{GRF}}$, which combines both the generated feedback signal F and the initial query $Q_{0}$ to support more effective retrieval. Section 2.3.1 details the prompt design and feedback types considered in our experiments, while Section 2.3.2 describes alternative strategies for integrating the GRF signal to enhance retrieval performance (prompts are reported in Section 6 of the Supplementary Data, available as supplementary data at *Bioinformatics* online).

#### 2.3.1 GRF prompt design

Each GRF prompt includes three components: (i) the mention span *m*; (ii) the mention context, *s*, namely a sentence from D containing *m*; (iii) a text generation task, *t*, that is an instruction defining the type of feedback to generate. We investigate the following feedback types:

- *Entity definition*: The most expressive feedback type, introduced by Shlyk et al. (2024), which generates a context-aware definition of the entity mention *m*. This type of feedback aims to explicitly capture the meaning of the mention within a concise sentence and use it to enrich the query.
- *N-synonyms*: Proposed by Dobbins (2024), this feedback generates a list of *n* exact synonyms or alternative phrasings for the given mention. The aim is to capture the variety of surface forms associated with a mention and address lexical mismatches between the mention span and concept names.
- *Standard name*: A new feedback type introduced in this study. The language model is prompted to generate a standard community-recognized name for the entity mention, conditioned on domain-specific terminology. This task is conceptually similar to zero-shot normalization, but instead of predicting a concept identifier, the model generates a standard name for the concept representing the entity mention. The generated name is not used as the final answer; instead, it serves as feedback to enrich the mention span during candidate retrieval. The domain-specific terminology used to condition the name generation task corresponds to the normalization target (e.g. NCBI Gene, NCBI Taxonomy, etc.).

#### 2.3.2 Integration strategies

**Figure 1:**
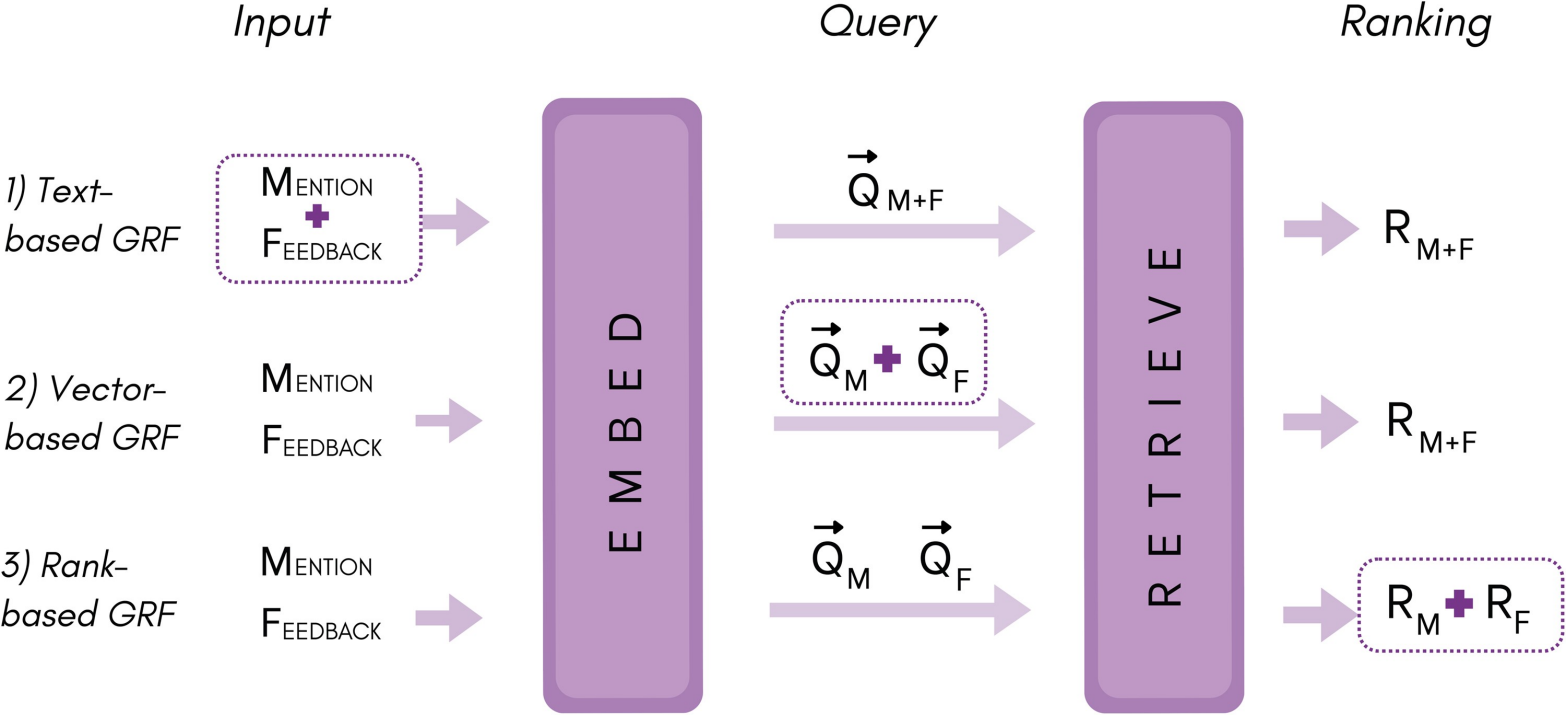
We illustrate three different strategies for integrating GRF signal into the candidate retrieval stage: (i) *Text-based GRF*: concatenates the mention span and the generated feedback text into a single string, (ii) *Vector-based GRF*: combines the mention and feedback vectors post-embedding, and (iii) *Rank-based GRF*: treats the mention span and feedback as two separate queries and fuses their candidate rankings, $R_{M}$ and $R_{F}$, post retrieval.

Depending on when the integration occurs, we distinguish three approaches for combining the GRF signal with the mention query $Q_{0}$ (see Fig. 1):

**Text-based GRF** integrates the GRF signal at the source by concatenating the original mention span *m* with the feedback text *F* into a single string, which is then processed by the embedding model. The embedding of this combined string is used to query the index, resulting in a candidate ranking R.

**Vector-based GRF** combines the vector representations of the generated feedback and the mention span post-embedding. Specifically, we embed the mention and feedback texts separately, then interpolate their embeddings using the Rocchio approach (Li et al. 2021a), as shown in Equation (1). The hyperparameter α controls the relative importance of the initial mention vector $Q_{0}$ with respect to the feedback vector $Q_{F}$, allowing the GRF signal to contextualize the mention vector in a tunable way (while all feedback types are evaluated with α=0.5 in our main experiments, Section 4.1.1 provides a detailed analysis of the effect of varying α across different feedback types.). For text-generation tasks that produce multi-vector feedback, as in the case of *n-synonyms*, we average *n* synonym embeddings before interpolating their aggregated vector representation with the mention vector. Our experiments showed that omitting this aggregation step significantly decreases the performance.


(1) $$\vec{Q_{\text{GRF}}}=\alpha \cdot \vec{Q_{0}}+(1-\alpha )\cdot {\vec{Q}}_{F}.$$

**Rank-based GRF** issues separate queries for the feedback and the mention span, obtaining independent candidate lists $R_{0}$ and $R_{F}$. These lists are then combined into a single ranking using a weighted variant of reciprocal rank fusion (RRF) method introduced in Mackie et al. (2023b). RRF scores a candidate by aggregating the reciprocals of its rank, r(c), in the individual ranked lists, as shown in Equation (2). Similar to the vector-based GRF, we use a hyperparameter α to control the contribution of each candidate ranking depending on its source. For the *n-synonym* feedback type, we aggregate all synonym embeddings into a single feedback vector before retrieval, ensuring that the generated feedback contributes only one candidate list to the RRF score. Alternatively, each synonym embedding can be treated as a separate query, contributing its own candidate list. We tested both strategies and found that the former often yields slightly better results; thus, we adopt it in the rank-based fusion approach.


(2) $$\text{RRF}(c)=\sum_{r\in \{R_{0},R_{F}\}}\frac{1}{r(c)}\times \{\begin{array}{cc} \alpha , & \text{if}\;r\in R_{0}\\ (1-\alpha ), & \text{if}\;r\in R_{F} \end{array}.$$

#### 2.3.3 All-in-one GRF

We examine the synergistic effect of combining different types of LLM-generated feedback for both vector- and rank-based GRF, which we refer to as *All-in-One* GRF. To incorporate multiple feedback signals, we extend the previous formulations as follows. For vector-based GRF, we use $\alpha =\frac{1}{\left(n+1\right)}$ for n feedback types, effectively averaging over the original and each feedback type. For the rank-based All-in-One approach, each type of generative feedback is treated as a separate query. The ranked lists produced by each feedback type and by the original mention are then combined with equal weight in the RRF score, regardless of their source ranking.

## 3 Experimental setup

### 3.1 Datasets and knowledge bases

All experiments in this study were conducted using corpora from BELB (Garda et al. 2023), a benchmark for standardized evaluation of models for BEL. Dataset selection was based on both relevance and public accessibility. Thus, corpora linked to KBs requiring restricted access, such as MedMentions, which is linked to UMLS and subject to a Data Usage Agreement, were excluded. We ultimately selected eight datasets linked to four publicly available KBs, enabling a comprehensive evaluation across representative entity types in molecular biology and genomics: diseases, chemicals, genes, and species. Each entity type presents distinct challenges for normalization (e.g. disease and chemical mentions suffer from high synonymy and abbreviation use; gene mentions are confounded by homology and overlapping nomenclature across organisms; and taxonomic names for species lack formal textual definitions). We retrieved the latest available versions of each KB and filtered the corpora to exclude entity mentions whose Concept Unique Identifiers (CUIs) were no longer present in the corresponding KB, due to curation decisions to obsolete or replace a concept (i.e. out-of-vocabulary [OOV] mentions). Following prior work, for NCBI Gene, we used KB subsets specific to the species of genes annotated in the GNormPlus and NLM-Gene corpora (for the detailed partitioning of NCBI Gene for each dataset see Table 1, available as supplementary data at *Bioinformatics* online). Descriptive statistics for the selected datasets and KBs are provided in Tables 1 and 2, respectively. For their detailed description, see Section 1 in Supplementary Data, available as supplementary data at *Bioinformatics* online.

**Table 1:** Overview of the corpora in BELB with number of documents and mentions in train/dev/test splits.

Entity type						
Corpus	Documents (train/dev/test)	Mentions (train/dev/test)	Composite	OOV	0-shot mentions	0-shot concepts
Disease						
NCBI Disease (Doğan et al. 2014)	592/100/100	5134/787/960	15 (1.56%)	2 (0.21%)	206 (21.84%)	80 (8.33%)
BC5CDR(D) (Li et al. 2016)	500/500/500	4182/4244/4424	53 (1.20%)	82 (1.85%)	648 (15.10%)	226 (5.10%)
Chemical						
BC5CDR(C) (Li et al. 2016)	500/500/500	5204/5348/5385	4 (0.07 %)	318 (5.91%)	467 (9.22%)	331 (6.14%)
NLM-Chem (Islamaj et al. 2022)^a^	80/20/50	21 218/5349/11 772	460 (3.91%)	329 (2.79%)	959 (8.73%)	607 (5.15%)
Species						
Linnaeus (Gerner et al. 2010)^a^	50/-/45	2194/-/2065	0 (0%)	4 (0.19%)	181 (8.78%)	155 (7.52%)
S800 (Pafilis et al. 2013)	500/-/125	2793/-/915	0 (0%)	0 (0%)	284 (31.03%)	225 (24.59%)
Gene						
GNormPlus (Wei et al. 2015)	277/-/254	3029/-/3223	57 (1.77%)	17 (0.53%)	1018 (32.32%)	994 (31.56%)
NLM-Gene (Islamaj et al. 2021)	400/50/100	11 266/1373/2728	205 (7.51%)	9 (0.33%)	639 (25.41%)	578 (22.99%)

We also report the number of composite and out-of-vocabulary (OOV) mentions in the test set, as well as the number of 0-shot mentions (number of de-duplicated mentions with no exact lexical overlap in the train/development set) and 0-shot concepts (number of de-duplicated mentions of unseen concepts, whose CUI is not present in the train/development set). Pairing of corpora and KB is determined by the entity type.

a Indicates full text corpus as opposed to abstract-based dataset.

**Table 2:** Overview of the KBs according to their entity type.

Entity type				
KB	Concepts	Names	Homonyms	Avg. names per concept
Disease				
CTD Diseases (Davis et al. 2023)	13 316	90 590	1885 (2.08%)	6.80
Chemical				
CTD Chemicals (Davis et al. 2023)	179 336	460 929	8 (0.00%)	2.57
Species				
NCBI Taxonomy (Scott, 2012)	2 650 584	3 106 735	5429 (0.00%)	1.17
Gene				
NCBI Gene (Brown et al. 2015)	42 252 923	105 570 090		
GNormPlus subset	843 978	2 190 640	372 487 (17.00%)	2.74
NLM-Gene subset	1 030 162	2 600 098	448 849 (17.26%)	2.65

We report the number of concepts, names, homonyms and average names per concept.

### 3.2 Baseline models

To demonstrate that GRF is a generalizable solution that can be used to enhance any BEL method that relies on the candidate retrieval for entity normalization, we use the following baselines:

**BioSyn** (Sung et al. 2020), a state-of-the-art name-based normalization system that adopts a hybrid approach, combining lexical features from sparse retrieval with learned dense vector representations. BioSyn is trained using synonym marginalization, that maximizes the similarity between a mention embedding and the embeddings of all surface forms associated with its gold concept in the KB. We fine-tune BioSyn separately on each dataset using its official training and development splits (for training details see Section 4 in Supplementary Data, available as supplementary data at *Bioinformatics* online).

**SapBERT** (https://huggingface.co/cambridgeltl/SapBERT-from-PubMedBERT-fulltext) (Liu et al. 2020), a biomedical embedding model pre-trained with a self-alignment objective to map entity mentions to their aliases in UMLS. We use SapBERT as an example of the off-the-shelf embedding model, and leverage publicly released pre-trained weights without additional fine-tuning.

### 3.3 Generative LLMs

For the main experiments, we rely on the OpenAI GPT-4o model for feedback generation, accessed programmatically via an API. In Section 4.2, we investigate whether smaller models can approximate the quality of feedback produced by larger, more costly LLMs. Specifically, we test two open-source model families from leading GenAI providers: Google’s Gemma-3 (Gemma Team Aishwarya Kamath et al. 2025) and Alibaba’s Qwen-3 (Bai et al. 2023), considering a range of sizes: 12/14B, 4B, 1B parameters. Inference for these models is performed on a Tesla H100 GPU.

### 3.4 Evaluation protocol

Tutubalina et al. (2020) demonstrated that evaluations based on official test sets tend to significantly overestimate the performance of entity linking systems. The reason is that a considerable portion of test mentions are either textual duplicates of other mentions within the test set or overlap with mentions in the training data. To get more realistic estimates of model performance, they proposed a fair evaluation protocol in which test sets are refined to include only zero-shot mentions, that is, mentions unseen during training. In this study, we adopt their protocol and use their preprocessing scripts to refine the test sets in the corpora used for our experiments. In addition, we remove all mentions linked to multiple concepts in the KB (*composite mentions*), since they do not allow for a unique linking decision and often arise from corpus-specific curation choices (for further discussion, see Section 2 in Supplementary Data, available as supplementary data at *Bioinformatics* online). All experiments are conducted on these refined test sets.

Model predictions are evaluated using recall@k, which directly reflects the effectiveness of the candidate-generation stage and provides an upper bound on end-to-end linking performance. It measures the proportion of test mentions for which the correct concept appears within the top-*k* ranked predictions. Because we are eventually interested in leveraging these predictions as input to a more computationally expensive reranker model, we focus on how the GRF mechanism affects recall at smaller cutoff values, k∈{1,2,3,5,10}, where recall@1 corresponds to accuracy.

## 4 Results

### 4.1 Evaluating GRF effectiveness for candidate retrieval

We evaluated the performance of GRF-enhanced systems against the baseline that relies on the original mention span for candidate retrieval. Table 3 reports the results for each individual feedback type and for the *All-in-One* GRF under the different integration strategies described in Section 2.3.2. Recall curves in Fig. 2 of the Supplementary Data, available as supplementary data at *Bioinformatics* online illustrate the effect of GRF at different cutoffs. For the *n -synonyms* feedback, we tested n∈{1,3,5,10}. For each value of *n*, we report the mean over five runs using distinct samples of LLM-generated synonyms. To simplify the presentation, Table 3 shows results only for *10-synonyms*; for the full sweep of n∈{1,3,5,10}, see Table 5 of the Supplementary Data, available as supplementary data at *Bioinformatics* online. We used the *10-synonyms* configuration in combination with *definition* and *standard name* feedback in the *All-in-One* approach, as it yielded the best performance (discussed further below). Unless otherwise stated, all standalone generative feedbacks were applied with α=0.5, giving equal weight to the feedback signal and the original entity mention. Sensitivity to α is discussed in Section 4.1.1.

**Table 3:** GRF performance with BioSyn (upper) and SapBERT (bottom) on refined test sets.

	CTD Diseases				CTD Chemicals				NCBI Gene				NCBI Taxonomy			
	(Disease)				(Chemical)				(Gene)				(Species)			
	NCBI Disease		BC5CDR (D)		BC5CDR (C)		NLM-Chem		GNormPlus		NLM-Gene		S800		Linnaeus	
	R@1	R@5	R@1	R@5	R@1	R@5	R@1	R@5	R@1	R@5	R@1	R@5	R@1	R@5	R@1	R@5
**Baseline—BioSyn**	71.35	85.92	74.69	87.19	82.01	88.65	70.90	79.87	69.15	85.26	33.80	78.87	61.61	75.00	69.06	86.74
+ Text-based—*10* synonyms	56.01	79.90	49.96	75.64	45.91	68.13	37.45	60.85	52.43	77.26	26.66	61.53	22.95	39.92	23.20	44.86
+ Text-based—definition	63.10	82.03	70.83	87.34	81.37	94.21	61.00	82.48	68.95	86.05	25.66	61.03	64.78	76.05	70.71	88.39
+ Text-based—standard name	64.07	85.43	71.60	86.72	89.72^a^	96.57	70.49	83.94	70.62	86.44	37.24	79.81	73.59	84.50	75.13	91.71
+ Vector-based—*10* synonyms	**73.78** ^a^	88.64	**78.51** ^a^	90.86	89.07	94.60	76.37^a^	85.35	74.55^a^	88.89	36.08	81.00	68.30	80.63	78.89^a^	90.93
+ Vector-based—definition	71.35	85.92	77.00	90.43	87.15	94.43	74.97	84.67	73.47	87.72	38.18	79.49	70.42	80.63	76.24	93.37
+ Vector-based—standard name	67.96	85.92	74.53	90.27	87.58	96.78	72.26	86.54	74.16	88.80	38.65 ^a^	82.15	75.70 ^a^	84.85	77.90	94.47^a^
+ Vector-based—*All-in-One*	72.81	86.79	76.57	91.35	**93.87**	97.43	**77.53**	88.32	78.76	91.27	**39.28**	82.87	**77.11**	85.56	**84.30**	95.58
+ Rank-based—*10* synonyms	71.35	89.32	73.61	91.23	84.02	95.28	71.69	85.56	72.71	90.27	35.08	81.37	64.08	82.04	74.80	90.60
+ Rank-based—definition	70.87	87.37	74.38	90.27	83.94	95.71	71.32	85.50	71.80	89.29	35.99	78.09	66.19	81.69	75.13	92.81
+ Rank-based—standard name	69.41	90.29 ^a^	74.22	91.35 ^a^	84.58	96.78^a^	72.26	86.96^a^	74.55^a^	91.55^a^	36.61	**85.13** ^a^	69.36	86.61 ^a^	76.24	93.92
+ Rank-based—*All-in-One*	71.65	**90.48**	77.25	**91.97**	93.27	**97.55**	77.22	**88.57**	**79.29**	**93.73**	37.77	83.00	75.14	**87.32**	83.64	**95.80**
**Baseline—SapBERT**	65.53	83.00	70.98	83.02	81.37	86.93	63.60	73.40	55.00	77.01	26.13	50.39	53.87	68.66	63.53	81.76
+ Text-based—*10* synonym	39.12	61.06	36.29	62.56	33.06	57.04	24.81	48.96	26.69	57.52	15.27	38.99	33.30	54.01	30.71	55.13
+ Text-based—definition	57.76	78.15	64.04	81.63	74.73	90.14	55.78	75.80	49.11	76.71	28.16	53.05	60.91	74.64	71.27	90.60
+ Text-based—standard name	61.16	77.66	67.90	81.17	87.36	95.28	66.73	80.81	49.60	79.46	26.60	53.52	67.25^a^	80.28	71.82	92.26
+ Vector-based—*10* synonyms	**67.66** ^a^	85.24	73.85 ^a^	84.96	87.40	93.14	70.30	79.31	57.81	82.27	28.73	53.86	63.09	75.14	75.02	89.28
+ Vector-based—definition	67.47	83.49	72.68	84.41	86.08	91.64	67.15	78.83	59.62	81.43	29.26^a^	54.77	63.73	76.40	75.69^a^	91.16
+ Vector-based—standard name	63.10	83.49	70.83	84.87	88.43^a^	96.14	72.05^a^	84.15^a^	55.00	83.10	29.26^a^	54.46	65.84	80.63	74.03	93.37
+ Vector-based—*All-in-One*	67.47	82.33	**73.95**	85.33	**92.93**	96.74	**74.78**	83.85	58.15	84.57	28.79	58.52	**70.49**	81.61	**80.44**	95.02
+ Rank-based—*10* synonyms	63.59	85.92	70.46	84.81	81.92	94.98	64.79	81.10	58.86	85.28	27.66	56.49^a^	57.67	75.21	70.27	89.06
+ Rank-based—definition	66.01	84.46	71.45	85.33	83.51	94.64	65.69	79.14	57.66	82.41	26.91	56.02	58.45	77.46	69.06	90.60
+ Rank-based—standard name	62.13	84.95 ^a^	70.52	**85.80** ^a^	83.08	96.78 ^a^	69.34	83.83	62.27 ^a^	85.95 ^a^	29.26 ^a^	55.71	59.15	80.98^a^	69.06	94.47^a^
+ Rank-based—*All-in-One*	65.72	**86.40**	73.61	85.67	92.84	**96.83**	74.05	**84.77**	**66.50**	**89.41**	**30.45**	**59.62**	68.66	**81.76**	78.89	**95.13**

**Bold** indicates best and underlined second-best results overall.

a Indicates the best standalone generative feedback type.

Our results show that incorporating GRF via either vector- or rank-based fusion consistently improves performance over the baseline, with effectiveness varying by feedback type. By contrast, text-based GRF is generally less effective and often underperforms the baseline. We attribute this to an “asymmetric query” effect: concatenating LLM-generated text with the mention span into a single long string produces a representation that diverges from the typically short target concept name, thereby increasing embedding-space distance and reducing retrieval quality. This “asymmetric query” effect is also evident with *definitions*, another long feedback type, compared to the shortest feedback type, that is, *standard name*, which suffers less from the length mismatch.

We find clear evidence of synergy when multiple feedback signals are used together to expand the original query: *All-in-One* GRF improves upon standalone feedback signals in nearly all benchmarks. While both integration strategies are effective, vector-based GRF, which incorporates LLM-generated feedback at the embedding stage, tends to offer the highest accuracy (Recall@1 gains of 1–15 percentage point). In contrast, rank-based fusion, which applies feedback post-retrieval, often achieves better performance for larger candidate sets, with Recall@5 gains of 4–12 percentage points.

Among individual feedback types, *standard name* provides the most effective standalone signal, especially in combination with rank-based fusion, attaining the highest recall for k>1 across all entity types and benchmarks. In Section 4.1.1 we further show that query expansion leveraging the *standard name* feedback can improve accuracy for suitable values of α. For *n -synonyms* feedback, deeper expansion (5–10 synonyms) generally outperforms shallower expansion (1–3 synonyms).

Although the fine-tuned BioSyn model outperforms SapBERT in absolute terms, the effect of GRF is consistent across both systems, underscoring the robustness of our findings. For an additional discussion on gene normalization, see Section 5 of the Supplementary Data, available as supplementary data at *Bioinformatics* online.

#### 4.1.1 Ablation study

To study the impact of weighting between query components in vector- and rank-based integration methods, we varied the mixing coefficient α over the range {0,1}. As described in Section 2.3.2, α controls the relative contribution of the original mention span *m* with respect to the LLM-generated feedback F. Setting α=0 corresponds to retrieval using only the generative feedback (i.e. without the original mention text), while α=1 reduced to the baseline configuration, in which the query consists solely of the mention span. Intermediate values interpolate between these two extremes, allowing us to assess how the interplay between *m* and F affects retrieval performance.

We find that across most feedback types, assigning approximately equal weight to the LLM-generated feedback and the original mention span yields the best performance (see Figs. 4 and 5 in Supplementary Data, available as supplementary data at *Bioinformatics* online, showing how the performances across feedback types change with α.) A notable exception is *standard name* feedback: when integrated via vector-based GRF, it achieves peak accuracy (Recall@1) at α≈0.6, outperforming other feedback types in 6 of 8 datasets. This positions *standard name* as a particularly strong signal for query expansion, capable of delivering both high recall (as shown in Section 4.1) and highly accurate top-1 matches for direct linking predictions when tuned appropriately. We note, however, that in our main experiments, we deliberately avoid hyperparameter tuning to ensure that all feedback types are evaluated under identical conditions.

Additionally, our analysis reveals a consistent performance gap in Recall@1 between the two integration methods, with rank-based GRF falling short of the accuracy achieved with vector-based GRF. For the *synonyms* and *definition* feedback categories, the largest gap occurs when α<0.5, whereas for the *standard name* category, the gap widens when α>0.5. Although the difference decreases rapidly at higher cutoff values (i.e. k>1), vector-based GRF remains the preferred approach when top-1 accuracy is the primary objective.

### 4.2 Sensitivity of GRF to the generative LLM

**Figure 2:**
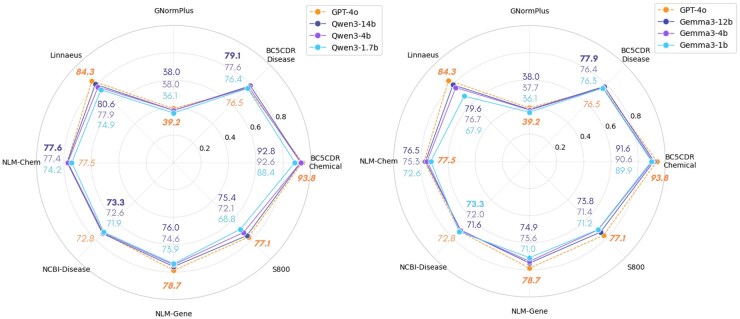
Comparison of retrieval accuracy using feedback generated by the Qwen-3 (left) and Gemma-3 (right) open-source model families, with GPT-4o feedback shown as a gold dashed line. Within each model family, a graded color scheme denotes model size, ranging from dark blue (models with >10B parameters) to light blue (models with 1B parameters). Best values for each datasets are highlighted in bold. Retrieval performance is evaluated using fine-tuned BioSyn under the vector-based *All-in-One* GRF strategy.

To assess how the choice of generative model affects GRF effectiveness, we evaluate a spectrum of model sizes from two leading families of open-source LLMs: Qwen-3 (14 b, 4 b, 1.7 b) and Gemma-3 (12 b, 4 b, 1 b). Figure 2 displays the retrieval accuracy (Recall@1) obtained with feedback from the open-source models, along with the results based on GPT-4o feedback (gold dashed line). All radar plots report retrieval performance across datasets under the vector-based *All-in-One* GRF. Per-feedback-type results are provided in Fig. 3 of the Supplementary Data, available as supplementary data at *Bioinformatics* online.

We find that smaller open-source LLM can generate feedback of comparable quality, often matching, and occasionally surpassing, the results obtained with the GPT-4o-based feedback, particularly in common biomedical domains such as Disease and Chemical. However, in more specialized domains with substantially larger term spaces, such as Gene and Species, performance differences become more noticeable, with GPT-4o retaining a clear advantage. Despite substantial differences in size, the performance gap between language models with >10B parameters and lightweight models with only 1B parameters remains relatively modest across most entity types (typically 1–4 percentage points), with the exception of Species, where it exceeds 5 percentage points. The larger gap in this domain relates to the specific feedback type used to enhance the query. A per-feedback-type analysis reveals that the *standard name* category is the most sensitive to model size, exhibiting larger performance drops with smaller generative models in terminology-intensive domains (see Fig. 4 in Supplementary Data, available as supplementary data at *Bioinformatics* online). *Synonyms* and *Definition* feedback categories, on the other hand, are more robust to smaller model size. This sensitivity likely reflects the greater knowledge demands of the former generative task, which requires the LLM to accurately recall domain-specific scientific terms leveraging its parametric knowledge. Smaller models may struggle to reproduce fine-grained scientific terminology reliably due to their limited exposure and greater terminological complexity in some domains. Taken together, these results suggest that GRF can deliver strong retrieval performance even when relying on smaller and more affordable generative models, but exhibits domain-specific sensitivity tied to the complexity of the underlying feedback generation task. Larger LLMs therefore offer clear benefits for more terminology-intensive entity types (e.g. Gene and Species), while smaller open-source models remain viable and effective for more common biomedical domains (e.g. Disease and Chemical).

### 4.3 Application of GRF-enhanced retrieval in cascading normalization pipelines

To demonstrate the value of high-recall candidate sets for end-to-end normalization, we present a practical use case that applies GRF-informed retrieval within a RAG-based normalization pipeline. In this setup, the output of the candidate generation stage serves as input to an LLM-based re-ranker, which is prompted to identify the most appropriate concept for a given mention based on its contextual information. We use OpenAI’s GPT-4o as the re-ranking model, experimenting with candidate sets of varying sizes (k∈{2,3,5,10}). As benchmarking re-ranking strategies lies beyond the scope of this study, we adopt an established prompting approach from recent work on RAG-based normalization (for additional details, see Section 7 of the Supplementary Data, available as supplementary data at *Bioinformatics* online).

Table 4 presents normalization results for two approaches: a two-stage RAG-based method and a simpler normalization strategy that uses the top-1 retrieved concept as the final linking prediction (*Peak top-1*). Candidate lists are generated using the *All-in-One* vector-based GRF strategy with fine-tuned BioSyn, which demonstrated strong performance in Section 4.1. The performance differences reported in parentheses quantify the relative improvements attributable to the GRF-enhanced candidate lists compared to the normalization based on plain retrieval. Overall, candidate lists obtained through GRF lead to consistent performance gains over the GRF-free alternative for both *Peak top-1* approach and the LLM-based re-ranking. For the latter, we observe that on several datasets the effect of GRF-enhanced retrieval diminishes as the number of in-context candidates increases. This pattern is consistent with our earlier findings: because GRF already achieves high recall at low *k*—ranking relevant candidates at the top—further enlarging the candidate list can introduce noise and hamper the LLM’s linking accuracy. In contrast, in the GRF-free setting, reranker can benefit from larger candidate list to access better candidate options. As a result, increasing the number of in-context candidates in the GRF-free setting generally improves normalization performance. In GRF-enabled settings, however, smaller candidate lists (3–5) often perform similarly or even better than larger lists, offering a more efficient option given token and computation costs.

**Table 4:** Normalization results for (i) the two-stage RAG-based approach with LLM-based re-ranking using candidate sets of varying sizes (k∈{2,3,5,10}) and (ii) a simpler linking approach that directly selects the top-1 retrieved concept (*Peak top-1*).

	*Pick top-1*	*LLM/w 2 cand.*	*LLM/w 3 cand.*	*LLM/w 5 cand.*	*LLM/w 10 cand.*
**Disease**					
NCBI disease	74.75 (+3.4)	75.72 (+3.88)	75.72 (+2.42)	74.75 (+1.48)	76.21 (+3.4)
BC5CDR (Disease)	76.69 (+2.0)	79.01 (+3.4)	79.78 (+4.01)	79.32 (+3.24)	79.32 (+2.16)
**Chemical**					
BC5CDR (Chemical)	94.00 (+12.0)	93.36 (+10.92)	93.79 (+8.78)	93.36 (+7.5)	92.71 (+6.63)
NLM-Chem	77.58 (+6.68)	79.77 (+6.78)	81.85 (+8.24)	82.06 (+6.99)	82.58 (+6.88)
**Gene**					
GNormPlus	78.68 (+9.53)	82.22 (+7.18)	82.71 (+4.72)	84.57 (+3.93)	85.26 (+3.34)
NLM-Gene	39.12 (+5.32)	61.34 (+4.85)	66.19 (+7.67)	67.44 (+3.13)	69.17 (+3.45)
**Species**					
S800	76.05 (+14.44)	77.46 (+11.62)	76.76 (+11.27)	75.00 (+7.75)	75.00 (+6.7)
Linnaeus	83.42 (+14.36)	90.60 (+9.94)	89.50 (+6.08)	91.16 (+7.19)	91.16 (+5.53)

For both methods, candidate lists are obtained using the *All-in-One* vector-based GRF strategy with fine-tuned BioSyn. Performance gains in parentheses are reported relative to the normalization using candidate lists obtained without GRF.

Comparing the two normalization methods, LLM-based re-ranking yields substantially larger gains than normalization based solely on top-1 retrieval, particularly for Gene and Species entity types. The joint effect of GRF-informed retrieval and LLM-based re-ranking leads to accuracy increases from 9% to 16% on GNormPlus, from 5% to 35% on NLM-Gene, and form 14% to 22% on Linnaeus, using 10 in-context candidates. On some datasets, however, the simpler top-1 approach remains competitive. For example, in BC5CDR (Chemical), GRF-enhanced retrieval alone delivers a 12% increase in top-1 accuracy over the baseline, reaching 94% accuracy at rank 1. These findings highlight the value of combining high-quality GRF-based candidate retrieval with advanced re-ranking methods to enhance end-to-end normalization performance. By plotting the results achieved with RAG-based normalization against the potential recall enabled by GRF at varying cutoffs (see Fig. 6 in the Supplementary Data, available as supplementary data at *Bioinformatics* online), we observe that there is still a margin for improvement left, suggesting the potential for even greater gains with more powerful re-ranking models.

## 5. Discussions

Our experiments demonstrate that GRF is a highly effective technique with great potential to advance biomedical entity linking applications. When properly integrated, GRF-enhanced candidate generation not only improves the accuracy of direct linking predictions but also provides a strong foundation for more advanced re-ranking methods by achieving high recall even with small candidate sets. Importantly, we found that GRF can also be effectively implemented with smaller open-source LLMs, maintaining competitive performance while reducing computational costs. However, we note that depending on the complexity of the feedback type some generative tasks may benefit from larger LLMs. By evaluating GRF with both, state-of-the-art BEL systems and off-the-shelf embedding models, we show that its benefits are robust and not tied to a specific retrieval system, suggesting broad applicability of this approach.

Based on an extensive analysis of different feedback types and integration strategies, we identify the following key observations: (i) incorporating the feedback signal at the embedding stage (*vector-based GRF*) yields the most effective candidate retrieval compared to text-based and rank-based fusion methods. This strategy achieves the highest retrieval accuracy and thus can be especially beneficial for direct linking prediction based on top-1 retrieval results; (ii) compounding feedback signals from multiple generative tasks can substantially improve retrieval performance over individual feedback signals. However, in practical applications, generating multiple feedback signals for a single mention is often impractical due to increased execution time, making a single strong feedback signal preferable; (iii) leveraging LLM-based knowledge of target terminology to generate standardized entity names provides the strongest standalone feedback signal, particularly when combined with the original mention span. We emphasize that the results reported in the main experiments were obtained without hyperparameter tuning. Additional gains are likely achievable through feedback-specific parameter optimization, as discussed in Section 4.1.1. In addition to its effectiveness, this feedback type is also computationally efficient, as the standardized names typically require fewer output tokens than definition- or synonym-based feedback generation tasks. However, due to the sensitivity issues discussed in Section 4.2, its use warrants additional caution.

Finally, while GRF-enhanced retrieval provides a clear advantage for cascading BEL pipelines, effectively mitigating the bounded recall problem, its full potential cannot be realized without powerful re-ranking methods. Our results in Section 4.3 show that, despite substantial gains, current frontier LLM-as-rerankers are not yet able to fully capitalize on high-recall candidate sets. We therefore encourage future work on the development and evaluation of improved re-ranking strategies.

## 6 Conclusion

We present a systematic evaluation of the GRF mechanism aimed at identifying effective ways to leverage GRF in BEL systems. Our evaluation explores multiple dimensions, including the analysis of different types of generative feedback and strategies for their integration. Through experiments conducted on eight biomedical corpora and four knowledge bases, we demonstrate that targeted text generation using LLMs can substantially enhance the effectiveness of the candidate generation stage, thereby improving the entity linking performance for biomedical text-mining applications. Beyond empirical findings, we provide practical recommendations to maximize the benefit of GRF for enhanced candidate retrieval. In addition to benchmarking recent GRF approaches, our study contributes a novel feedback generation strategy that delivers strong standalone signal for query expansion, while remaining highly cost-effective. Finally, we show that GRF not only improves direct linking accuracy but also produces high-quality candidate sets that can significantly benefit downstream re-ranking models. We encourage future work to investigate re-ranking methods that can fully realize the potential of GRF in end-to-end BEL pipelines.

## Supplementary Material

btag011_Supplementary_Data

## Data Availability

Our code and data are available at: https://github.com/dash-ka/Biomedical-Entity-Linking-GRF or https://doi.org/10.5281/zenodo.17853541
