# Supplementary material for: Improving biomedical entity linking with generative relevance feedback
Source: Bioinformatics. 2026 Jan 14;42(2):btag011. doi: 10.1093/bioinformatics/btag011 (PMC12866626; doi:10.1093/bioinformatics/btag011)
Supplement: btag011_Supplementary_Data [file btag011_supplementary_data.pdf]

Journal Title Here, 2025, 1–8
doi: DOI HERE
Advance Access Publication Date: Day Month Year
Paper

Supplementary data for

Improving biomedical entity linking with generative relevance feedback

Darya Shlyk[1,*] and Lawrence Hunter[2]

[1]Department of Computer Science, Università degli Studi di Milano, Celoria 18, 20133, Milan, Italy and [2]Department of Pediatrics, The University of Chicago, 5801 South Ellis Avenue, Chicago, IL, 60637, USA

*Corresponding author. darya.shlyk@unimi.it.

FOR PUBLISHER ONLY Received on Date Month Year; revised on Date Month Year; accepted on Date Month Year

Abstract

Motivation: Biomedical Entity Linking (BEL) maps mentions in biomedical text to standardized identifiers, enabling structured data integration and downstream knowledge discovery. However, current BEL systems remain fundamentally constrained by the recall of the initial candidate pool, where suboptimal retrieval limits the overall effectiveness of the normalization pipeline.
Results: We present the first systematic evaluation of *Generative Relevance Feedback* (GRF) for enhancing candidate retrieval in state-of-the-art BEL systems. GRF leverages large language models (LLMs) to enrich the expressiveness of the mention in a zero-shot fashion. We assess GRF's impact under two scenarios—direct linking prediction and candidate generation in cascading normalization pipelines—and analyze its sensitivity to different LLMs, feedback types, and integration strategies. Experiments across eight corpora and four biomedical knowledge bases demonstrate that integrating GRF significantly improves both accuracy and recall, thereby increasing the upper bound on normalization performance. Our findings highlight GRF as an efficient, model-agnostic solution and underscore its potential as a key component for advancing BEL.
Availability: The code to reproduce our experiments can be found at: https://github.com/dash-ka/Biomedical-Entity-Linking-GRF.

1. Corpora and Knowledge Bases

In this section we describe in detail the corpora and knowledge bases, grouped by their entity type:

Gene For gene normalization, we use `GNormPlus` (Wei et al., 2015), an extension of the BioCreative II Gene Normalization (BC2GN) corpus (Morgan et al., 2008), re-annotated at the *mention level*. `NLM-Gene` (Islamaj et al., 2021) is a more recent benchmark that includes gene names across multiple species. Both corpora are linked to NCBI Gene (Brown et al., 2015). For each dataset, we construct a subset of NCBI Gene corresponding to the host species represented in that corpus. Table 2 presents the detailed partitioning of NCBI Gene for each dataset.

Disease We use the `NCBI Disease` corpus (Doğan et al., 2014) and `BC5CDR` (Li et al., 2016), both widely adopted benchmarks for disease normalization. `BC5CDR` was originally developed for the Chemical–Disease Relation (CDR) track at BioCreative V. Entity mentions in both corpora are normalized to CTD Diseases (Davis et al., 2023), also known as MEDIC, a curated ontology derived from the "Diseases" branch of MeSH (Lipscomb, 2000) and supplemented with genetic disorders from OMIM (Hamosh et al., 2005).

Chemical For chemical normalization, we use chemical annotations from `BC5CDR` and `NLM-Chem` (Islamaj et al., 2022). The latter is a manually curated collection of full-text biomedical articles released for the NLM-Chem track at BioCreative VII. Both corpora are normalized to CTD Chemicals (Davis et al., 2023), which integrates descriptors from the "Chemicals and Drugs" category of MeSH and Supplementary Concept Records.

Species For species normalization, we use the `Linnaeus` corpus (Martin et al., 2010), consisting of annotated full-text articles, and `S800` (Pafilis et al., 2013), containing 800 abstracts across eight publication categories (e.g., bacteriology, virology, zoology). Both corpora are normalized to NCBI Taxonomy (Scott, 2012), the standard hierarchical classification system of organisms used in GenBank and related sequence databases. As neither corpus includes predefined data splits, we randomly partition them into training and test sets.

© The Author 2025. Published by Oxford University Press. All rights reserved. For permissions, please e-mail: journals.permissions@oup.com

To characterize the difficulty of the linking task across the different corpora, Table 1 reports additional statistics for the evaluation sets, including the number of zero-shot test mentions and concepts after Tutubalina filtering, the rate of exact lexical overlap in the filtered test set, and the N-gram (N=3) Jaccard similarity between each test mention and its corresponding concept aliases. The N-gram Jaccard similarity is computed by taking the maximum similarity value across all aliases of the gold concept.

Entity type / Corpus	Test mentions	Exact overlap	Jaccard sim.	0-shot mentions	0-shot concepts
Disease					
`NCBI Disease` (Doğan et al., 2014)	960	0.19	0.48	206 (21.84 %)	80 (8.33 %)
`BC5CDR` (D) (Li et al., 2016)	4,424	0.36	0.59	648 (15.10 %)	226 (5.10 %)
Chemical					
`BC5CDR` (C) (Li et al., 2016)	5,385	0.62	0.75	467 (9.22 %)	331 (6.14 %)
`NLM-Chem` † (Islamaj et al., 2022)	11,772	0.40	0.59	959 (8.73 %)	607 (5.15 %)
Species					
`Linnaeus` † (Gerner et al., 2010)	2,065	0.06	0.42	181 (8.78 %)	155 (7.52 %)
`S800` (Pafilis et al., 2013)	915	0.10	0.39	284 (31.03 %)	225 (24.59 %)
Gene					
`GNormPlus` (Wei et al., 2015)	3,223	0.51	0.65	1,018 (32.32 %)	994 (31.56 %)
`NLM-Gene` (Islamaj et al., 2021)	2,728	0.46	0.64	639 (25.41 %)	578 (22.99 %)

Table 1. Statistics summarizing the difficulty of the entity linking task across evaluation corpora, including the number of zero-shot test mentions and concepts after Tutubalina filtering, exact lexical overlap, and trigram (N=3) Jaccard similarity between each mention and its true concept aliases.

2. Composite mentions

Multiple concepts with overlapping spans in a corpus may be annotated as a single composite mention. These typically arise from phrases that join distinct concepts using conjunctions (e.g., "or", "and") or punctuation (e.g., commas or dashes). In some cases, annotation guidelines explicitly define rules that give rise to ambiguous mentions. For instance, the `NLM-Chem` corpus guidelines state: "If the chemical is a mixture named as multiple chemicals, whose mentions are clearly separate, or are combined with a dash, annotate separate chemicals by assigning the MeSH ID for each ingredient that comprises that mixture." This rule produces highly ambiguous annotations such as "tetramethylrhodamine ethyl ester perchlorate", which is simultaneously linked to two concepts: "TMRE probe" (MESH:C110932) and "perchloric acids" (MESH:D010472). Similar ambiguities are also observed in the `NLM-Gene` corpus. These composite mentions cannot be resolved by BEL, since they do not permit a unique linking decision. Garda et al. (2023) propose retaining these mentions and applying a lenient evaluation strategy, where a prediction is considered correct if it matches any gold concept. However, such strategies risk overestimating the true performance of linking systems (Zhang et al., 2022). Because composite mentions generally result from corpus-specific curation decisions and represent only a minor fraction of test data, we argue that they should be addressed during the NER stage and post-processed prior to linking. To enable accurate evaluation of BEL models, we therefore exclude composite mentions from our test sets, without compromising the generalizability of our results.

3. Homonym Disambiguation

Biomedical knowledge bases (KBs) are rich in homonyms, identical names, or aliases, shared by distinct biomedical concepts. As discussed in Garda and Leser (2024), homonyms can severely limit the applicability of name-based BEL methods that link a mention span to the most similar concept name in the KB. It is immediately clear that such approaches fail when a name points to multiple concepts. Therefore, prior to constructing the index of unique concept names, we perform homonym disambiguation using the algorithm presented in Garda and Leser (2024). Specifically, we first collect all concept names that are homonyms in a given KB, then augment each name as follows: if the homonym is not the preferred name of the concept, we append the preferred name to the homonym; otherwise, we append another name associated with that concept that is not homonym. For example, "A2M" becomes "A2M (α2-microglobulin)". Unlike the original implementation, we prefer to use the longest non-homonym name for disambiguation rather than the shortest. Gene names require an additional layer of disambiguation, as the same gene symbol may refer to genes from different species (*cross-species homonyms*). For instance, in NCBI Gene, "A2M" denotes both a human and a cattle gene. To address this, we extend the standard homonym disambiguation procedure by appending the species name to each gene alias. For example, we generate two disambiguated gene names: "A2M (α2-microglobulin) (human)" and "A2M (α2-microglobulin) (cattle)". In contrast to Garda and Leser (2024), we apply cross-species disambiguation to all gene names in NCBI Gene, not solely to homonyms.

NCBI Taxonomy		
Identifier	Name	Corpus
3702	Arabidopsis thaliana	NLM-Gene, GNormPlus
438753	Azorhizobium caulinodans ORS 571	GNormPlus
81972	Arabidopsis lyrata subsp. lyrata	NLM-Gene
9913	Bos taurus	NLM-Gene, GNormPlus
6239	Caenorhabditis elegans	NLM-Gene, GNormPlus
9615	Canis lupus familiaris	NLM-Gene
10029	Cricetulus griseus	NLM-Gene
237561	Candida albicans SC5314	GNormPlus
3055	Chlamydomonas reinhardtii	NLM-Gene
7227	Drosophila melanogaster	NLM-Gene, GNormPlus
6956	Dermatophagoides pteronyssinus	NLM-Gene
7955	Danio rerio	NLM-Gene, GNormPlus
2886926	Escherichia phage P1	NLM-Gene
511145	Escherichia coli str. K-12 substr. MG1655	GNormPlus
9031	Gallus gallus	NLM-Gene, GNormPlus
3847	Glycine max	GNormPlus
9606	Homo sapiens	NLM-Gene, GNormPlus
11676	Human immunodeficiency virus 1	NLM-Gene, GNormPlus
11709	Human immunodeficiency virus 2	NLM-Gene
10376	Human gammaherpesvirus 4	GNormPlus
333760	Human papillomavirus 16	GNormPlus
39015	Human T-cell leukemia virus type I	GNormPlus
10359	Human betaherpesvirus 5	GNormPlus
10298	Human alphaherpesvirus 1	GNormPlus
10090	Mus musculus	NLM-Gene, GNormPlus
10089	Mus caroli	NLM-Gene
51031	Necator americanus	NLM-Gene
9940	Ovis aries	NLM-Gene
9986	Oryctolagus cuniculus	NLM-Gene, GNormPlus
10116	Rattus norvegicus	NLM-Gene, GNormPlus
9823	Sus scrofa	NLM-Gene, GNormPlus
559292	Saccharomyces cerevisiae S288C	NLM-Gene, GNormPlus
559292	Schizosaccharomyces pombe	NLM-Gene,
284812	Schizosaccharomyces pombe 972h-	GNormPlus
8355	Xenopus laevis	NLM-Gene, GNormPlus
8364	Xenopus tropicalis	NLM-Gene, GNormPlus

Table 2. NCBI Gene subsets determined by the species (NCBI Taxonomy entries) of the gene mentions in `GNormPlus` and `NLM-Gene`.

4. Training details

For **BioSyn**, we convert all corpora and KBs in the format required by the model and re-train it on each dataset with the default hyper-parameters reported by the original implementations. For training, we use both the train and development splits, when available. The backbone pre-trained language model is SapBERT, which prior studies showed superior to the original implementation using BioBERT Lee et al. (2019). The models are trained and evaluated using one Nvidia H100 GPU.

5. Effect of species assignment on gene normalization

Gene mentions pose significant challenges for normalization systems due to the inherent ambiguity introduced by the host organism. Determining the species to which a gene mention belongs is often necessary to link it to the correct gene identifier in the knowledge base. For example, NCBI Gene contains four distinct entries for *"interleukin 2"*: *interleukin 2 (Mus musculus)* `[16183]`, *interleukin 2 (Homo sapiens)* `[3558]`, *interleukin 2 (Rattus norvegicus)* `[116562]`, and *interleukin 2 (Ovis aries)* `[443401]`. The correct linking decision therefore depends on whether the gene mention refers to a human, mouse, rat, or sheep. Although contextual clues can provide species information, accurately detecting and associating species names with gene mentions remains a challenging task.

Considering the importance of host-species information for gene normalization, we analyzed the species distribution in both `GNormPlus` and `NLM-Gene` (see Figure 1) to better understand the discrepancy in normalization performance observed between the two datasets (Table 3, Section 4.1 in the main paper). We found that `GNormPlus` is largely homogeneous, with over 80% of gene mentions referring to human

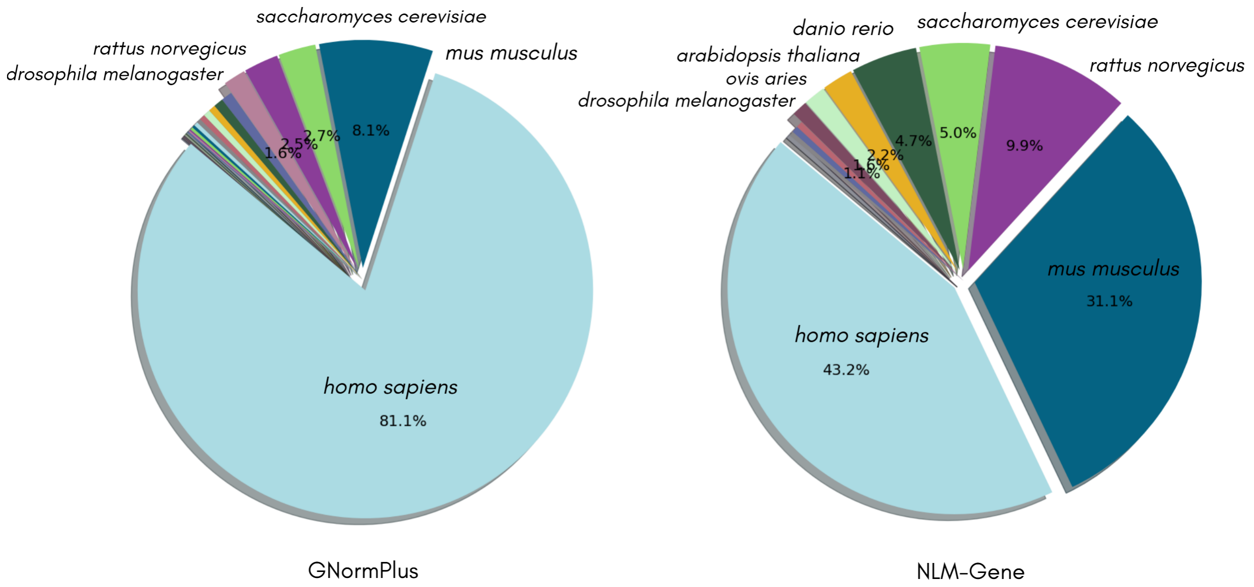

Fig. 1: Distribution of host-species across gene mentions in `GNormPlus` and `NLM-Gene` (test sets).

genes, whereas NLM-Gene is considerably more diverse: only about 40% of mentions are human, and the remaining 60% are distributed across other species (e.g. Mus musculus, Rattus norvegicus, and Saccharomyces cerevisiae, etc.). This higher species heterogeneity in NLM-Gene accounts for its lower normalization accuracy. When species information is missing from the gene mention, retrieval may surface the correct gene name across multiple organisms, but the model cannot make a reliable linking decision without explicit species information.

To address this issue, we experimented with applying the same disambiguation strategy used for KB homonyms (Section 3) to qualify gene mentions with the name of their host species (e.g., "ovalbumin (Mus musculus)"), and used these augmented mentions as queries for candidate retrieval. Table 3 compares the retrieval effectiveness of qualified gene mentions under two approaches: (1) *Qualified-SA*, which uses SpeciesAssignment (SA) (Luo et al., 2022), a deep learning model trained to assign species to genes via sequence labeling; and (2) *Oracle*, which uses the species name associated with the gold-standard gene to qualify the mention. The latter represents an ideal scenario in which species information is assumed to be perfectly accurate, allowing us to assess how species assignment errors in *Qualified-SA* might affect retrieval performance. Both approaches are compared against the unqualified baseline used in our main experiments.

The results show a substantial increase in accuracy on both datasets when using species-assigned gene mentions compared to the unqualified baseline. Recall@1 with the *Oracle* approach rises from 69.15 to 77.60 on `GNormPlus`, and from 33.80 to 69.79 on `NLM-Gene` (more than doubling the initial performance). These findings highlight the importance of using qualified mentions, particularly for cross-species gene normalization. The results for *Qualified-SA* are close to the ideal; however, we note that Recall@5 on the simpler dataset (`GNormPlus`) drops slightly below the baseline, suggesting caution when applying automated species assignment methods, as incorrectly assigned species may negatively affect downstream normalization performance.

	`GNormPlus`		`NLM-Gene`	
	R@1	R@5	R@1	R@5
Baseline	69.15	85.26	33.80	78.87
Qualified-SA	72.20	82.41	63.84	82.00
Oracle	77.60	87.81	69.79	83.56

Table 3. Comparison of normalization performance using species-qualified gene mentions (*Qualified-SA* and *Oracle*) versus unqualified mentions (*Baseline*)

We re-ran the experiments using GRF-enhanced retrieval on the qualified `GNormPlus` and `NLM-Gene` datasets, where species information was assigned to gene mentions via SpeciesAssignment (SA). Table 4 presents the evaluation results for both BioSyn and SapBERT baselines. Overall recall values improved across both datasets, but the main trends observed in our main experiments remain consistent. Notably, for the more challenging `NLM-Gene` dataset, while GRF continues to provide gains in vector-based retrieval, the primary driver of the observed accuracy improvement is the inclusion of explicit species information.

	NCBI Gene (Gene)			
	GNormPlus		NLM-Gene	
	R@1	R@5	R@1	R@5
**Baseline - BioSyn**	72.20	82.41	63.84	82.00
+ Text-based - *1* synonym	70.70	83.45	55.99	80.65
+ Text-based - *3* synonym	67.64	83.28	44.78	77.74
+ Text-based - *5* synonym	64.34	81.74	40.03	74.08
+ Text-based - *10* synonym	59.80	79.84	35.61	70.32
+ Text-based - definition	73.37	83.98	37.71	69.48
+ Text-based - standard name	75.14	87.03	59.78	87.79
+ Vector-based - *1* synonym	74.16	86.58	62.72	84.44
+ Vector-based - *3* synonyms	77.30	88.19	66.32	85.57
+ Vector-based - *5* synonyms	77.40	88.60	**67.07** †	85.69
+ Vector-based - *10* synonyms	77.46	88.58	66.94	85.85
+ Vector-based - definition	76.03	85.36	65.25	84.35
+ Vector-based - standard name	78.88 †	91.55	65.72	**89.82** †
+ Vector-based - *All feedback*	81.06	92.43	63.59	88.70
+ Rank-based - *1* synonym	72.22	87.30	62.25	84.16
+ Rank-based - *3* synonyms	74.55	89.43	63.25	86.16
+ Rank-based - *5* synonyms	75.16	90.55	63.53	86.32
+ Rank-based - *10* synonyms	75.36	90.90	63.53	86.79
+ Rank-based - definition	75.14	86.73	62.44	81.84
+ Rank-based - standard name	77.21	91.74 †	63.06	88.10
+ Rank-based - *All feedback*	**81.51**	**94.04**	59.87	88.32
**Baseline - SapBERT**	67.58	77.11	61.97	75.58
+ Text-based - *1* synonym	56.77	77.46	56.21	76.21
+ Text-based - *3* synonym	44.36	71.17	44.03	70.04
+ Text-based - *5* synonym	37.83	67.26	38.24	65.82
+ Text-based - *10* synonym	34.00	63.81	35.86	63.19
+ Text-based - definition	57.66	79.37	58.37	77.30
+ Text-based - standard name	62.67	81.33	63.53	80.90
+ Vector-based - *1* synonym	69.01	82.88	62.53	79.15
+ Vector-based - *3* synonyms	71.19	83.96	**66.32** †	80.59
+ Vector-based - *5* synonyms	71.47	83.88	66.07	80.28
+ Vector-based - *10* synonyms	71.59	83.47	66.10	80.71
+ Vector-based - definition	70.82	81.43	65.41	79.49
+ Vector-based - standard name	74.36†	88.99†	62.75	82.62 †
+ Vector-based - *All feedback*	71.33	89.46	65.44	83.59
+ Rank-based - *1* synonym	65.61	82.04	59.93	78.34
+ Rank-based - *3* synonyms	67.81	85.22	59.62	80.06
+ Rank-based - *5* synonyms	68.72	86.40	60.68	81.03
+ Rank-based - *10* synonyms	69.39	87.07	59.74	81.81
+ Rank-based - definition	69.74	84.08	63.53	81.06
+ Rank-based - standard name	70.53	88.89	58.68	80.75
+ Rank-based - *All feedback*	**75.48**	**92.41**	61.37	**84.72**

Table 4. GRF performance on GNormPlus and NLM-Gene using species-qualified test mentions. **Bold** indicates best and underlined second-best results overall. † indicates the best standalone generative feedback type. Values below the baseline are colored in red.

6. GRF prompts

Below we list the prompts used to generate the feedback types discussed in Section 2.3.1: *n-synonyms*, *entity definition*, and *standard name*.

```
You are an expert biocurator.
Given the context: "{context}", specify twenty exact synonyms of the concept **{concept}**.
```

```
These should be either alternative names, morphological variants, or terminological
equivalents referring to the same underlying concept (not broader or related terms).
Return your answer as a JSON object in the following format:
{"synonyms": <exact synonyms here separated by a colon (e.g., "synonym1:synonym2:synonym3:synonym4:synonym5 etc.")>}
If the concept is an acronym, always expand it by including the full name in parentheses.

You are an expert biocurator.
Given the context: "{context}", specify a single sentence definition of the concept **{concept}**.
Return your answer as a JSON object in the following format:
{"definition": <"a single sentence definition of the concept here">}

You are an expert biocurator.
Given the context: "{context}", specify the standard concept name from {the target terminology}
for the concept **{concept}**.
Return your answer as a JSON object in the following format:
{"name": <"a standard community-recognized concept name here">}
```

7. Prompting strategy for LLM-based re-ranking

We build on the recent work of Dobbins (2024), who explored optimal prompting strategies for re-ranking candidate concepts. Specifically, we adopt their multiple-choice prompting approach, which offers a good balance between effectiveness and cost efficiency. This method presents all candidate concepts within a single prompt and instructs the LLM to identify the most appropriate concept for a given mention based on its context. Rather than listing candidate concepts by their preferred names, we use the surface form s that achieved the highest score among the retrieved candidate names in R^k. Each surface form is listed in the prompt alongside its corresponding concept identifier (e.g., "D001919": *bradyarrhythmia*). The expected model output consists of a list of concept identifiers selected for the mention. Contrary to the findings of Dobbins (2024), we observed that the chain-of-thought (CoT) prompting technique resulted in suboptimal normalization performance. Consequently, we employed a simpler prompt, which proved both more effective and more cost-efficient, as it generated fewer output tokens. The prompt template is shown below:

```
You are an expert biocurator. Your task is to map an entity mention to the correct concept.
You can use the context to disambiguate the meaning of the entity mention.
Context: {context}
Select which of the following concepts best represents the entity mention: {mention}.
Concepts:
{concepts}
If uncertain between two concepts keep both.
Format the output as the following JSON
{id: <a list of selected concept identifiers.>}
```

References

G. R. Brown, V. Hem, K. S. Katz, M. Ovetsky, C. Wallin, O. Ermolaeva, I. Tolstoy, T. Tatusova, K. D. Pruitt, D. R. Maglott, and T. D. Murphy. Gene: a gene-centered information resource at ncbi. *Nucleic Acids Research*, 43(D1):D36–D42, 2015. ISSN 0305-1048. doi: 10.1093/nar/gku1055.

A. P. Davis, T. C. Wiegers, R. J. Johnson, D. Sciaky, J. Wiegers, and C. J. Mattingly. Comparative toxicogenomics database (ctd): update 2023. *Nucleic Acids Research*, 51:D1257–D1262, 1 2023. doi: 10.1093/nar/gkac833.

N. J. Dobbins. Generalizable and scalable multistage biomedical concept normalization leveraging large language models. *ArXiv*, abs/2405.15122, 2024. URL https://api.semanticscholar.org/CorpusID:270045271.

R. I. Doğan, R. Leaman, and Z. Lu. Ncbi disease corpus: A resource for disease name recognition and concept normalization. *Journal of Biomedical Informatics*, 47:1–10, 2014. ISSN 1532-0464. doi: 10.1016/j.jbi.2013.12.006.

S. Garda and U. Leser. Belhd: improving biomedical entity linking with homonym disambiguation. *Bioinformatics*, 40, 2024. URL https://api.semanticscholar.org/CorpusID:266903053.

S. Garda, L. Weber-Genzel, R. Martin, and U. Leser. Belb: a biomedical entity linking benchmark. *Bioinformatics*, 39, 2023. URL https://api.semanticscholar.org/CorpusID:261064932.

M. Gerner, G. Nenadic, and C. M. Bergman. Linnaeus: A species name identification system for biomedical literature. *BMC Bioinformatics*, 11:85 – 85, 2010. URL https://api.semanticscholar.org/CorpusID:10197117.

A. Hamosh, A. F. Scott, J. S. Amberger, C. A. Bocchini, and V. A. McKusick. Online mendelian inheritance in man (omim), a knowledgebase of human genes and genetic disorders. *Nucleic acids research*, 33(suppl_1):D514–D517, 2005.

	CTD Diseases (Disease)				CTD Chemicals (Chemical)				NCBI Gene (Gene)				NCBI Taxonomy (Species)			
	NCBI Disease		BC5CDR (D)		BC5CDR (C)		NLM-Chem		GNormPlus		NLM-Gene		S800		Linnaeus	
	R@1	R@5	R@1	R@5	R@1	R@5	R@1	R@5	R@1	R@5	R@1	R@5	R@1	R@5	R@1	R@5
**Baseline - BioSyn**	71.35	85.92	74.69	87.19	82.01	88.65	70.90	79.87	69.15	85.26	33.80	78.87	61.61	75.00	69.06	86.74
+ Text-based - *1* synonym	62.33	82.42	64.50	85.52	69.46	85.69	58.70	78.14	64.91	84.20	34.11	75.36	63.80	77.11	64.64	84.64
+ Text-based - *3* synonyms	60.09	79.70	58.36	81.38	56.74	78.92	49.67	71.22	60.19	82.37	31.89	71.11	44.50	64.43	43.53	68.39
+ Text-based - *5* synonyms	56.79	80.38	55.18	79.35	49.29	72.59	41.33	64.50	55.93	79.41	29.51	65.66	27.95	48.52	28.61	54.91
+ Text-based - *10* synonyms	56.01	79.90	49.96	75.64	45.91	68.13	37.45	60.85	52.43	77.26	26.66	61.53	22.95	39.92	23.20	44.86
+ Text-based - definition	63.10	82.03	70.83	87.34	81.37	94.21	61.00	82.48	68.95	86.05	25.66	61.03	64.78	76.05	70.71	88.39
+ Text-based - standard name	64.07	85.43	71.60	86.72	89.72 †	96.57	70.49	83.94	70.62	86.44	37.24	79.81	73.59	84.50	75.13	91.71
+ Vector-based - *1* synonym	68.15	85.53	71.35	89.53	84.53	91.69	69.36	82.52	69.72	86.32	34.71	76.87	61.61	76.19	70.38	91.38
+ Vector-based - *3* synonyms	72.71	87.47	76.72	90.83	88.77	93.91	75.22	85.04	73.81	88.58	36.58	80.68	66.97	80.07	78.67	91.49
+ Vector-based - *5* synonyms	**73.78** †	88.15	77.77	90.77	88.99	93.91	75.51	85.15	74.53	88.56	36.43	80.78	67.81	80.21	78.89 †	92.04
+ Vector-based - *10* synonyms	**73.78** †	88.64	**78.51** †	90.86	89.07	94.60	76.37 †	85.35	74.55 †	88.89	36.08	81.00	68.30	80.63	78.89 †	90.93
+ Vector-based - definition	71.35	85.92	77.00	90.43	87.15	94.43	74.97	84.67	73.47	87.72	38.18	79.49	70.42	80.63	76.24	93.37
+ Vector-based - standard name	67.96	85.92	74.53	90.27	87.58	96.78	72.26	86.54	74.16	88.80	38.65 †	82.15	75.70 †	84.85	77.90	94.47 †
+ Vector-based - *All-in-One*	72.81	86.79	76.57	91.35	**93.87**	97.43	**77.53**	88.32	78.76	91.27	**39.28**	82.87	**77.11**	85.56	**84.30**	95.58
+ Rank-based - *1* synonym	68.32	87.47	70.33	89.84	80.85	91.56	68.55	82.62	69.80	87.46	34.27	78.62	61.61	79.29	70.27	89.50
+ Rank-based - *3* synonyms	70.19	88.34	71.57	90.89	83.38	94.21	70.59	84.54	71.59	89.31	34.96	80.90	62.53	80.63	72.70	90.27
+ Rank-based - *5* synonyms	70.67	88.83	73.14	91.20	83.72	94.68	71.69	85.19	72.10	89.78	35.39	81.03	64.43	81.33	73.59	91.16
+ Rank-based - *10* synonyms	71.35	89.32	73.61	91.23	84.02	95.28	71.69	85.56	72.71	90.27	35.08	81.37	64.08	82.04	74.80	90.60
+ Rank-based - definition	70.87	87.37	74.38	90.27	83.94	95.71	71.32	85.50	71.80	89.29	35.99	78.09	66.19	81.69	75.13	92.81
+ Rank-based - standard name	69.41	90.29 †	74.22	91.35 †	84.58	96.78 †	72.26	86.96 †	74.55 †	91.55 †	36.61	**85.13** †	69.36	86.61 †	76.24	93.92
+ Rank-based - *All-in-One*	71.65	**90.48**	77.25	**91.97**	93.27	**97.55**	77.22	**88.57**	**79.29**	**93.73**	37.77	83.00	75.14	**87.32**	83.64	**95.80**
**Baseline - SapBERT**	65.53	83.00	70.98	83.02	81.37	86.93	63.60	73.40	55.00	77.01	26.13	50.39	53.87	68.66	63.53	81.76
+ Text-based - *1* synonym	54.75	74.75	57.43	76.57	67.40	82.48	49.78	69.21	47.68	73.90	24.13	51.23	56.69	72.46	62.76	84.64
+ Text-based - *3* synonym	46.40	68.05	47.62	71.04	45.65	71.39	36.51	59.68	38.27	67.99	20.68	46.94	45.00	65.14	47.07	70.82
+ Text-based - *5* synonym	41.35	65.53	41.17	67.25	36.27	62.05	28.59	52.47	31.96	62.29	16.18	42.53	36.90	58.38	37.34	62.09
+ Text-based - *10* synonym	39.12	61.06	36.29	62.56	33.06	57.04	24.81	48.96	26.69	57.52	15.27	38.99	33.30	54.01	30.71	55.13
+ Text-based - definition	57.76	78.15	64.04	81.63	74.73	90.14	55.78	75.80	49.11	76.71	28.16	53.05	60.91	74.64	71.27	90.60
+ Text-based - standard name	61.16	77.66	67.90	81.17	87.36	95.28	66.73	80.81	49.60	79.46	26.60	53.52	67.25 †	80.28	71.82	92.26
+ Vector-based - *1* synonym	63.00	82.23	68.24	83.17	82.09	90.74	64.15	77.51	54.47	79.54	26.32	52.08	56.54	71.19	65.63	87.95
+ Vector-based - *3* synonyms	66.89	84.56	73.14	84.59	86.89	92.41	69.78	79.43	57.36	81.70	28.10	53.92	63.23	75.07	74.03	89.50
+ Vector-based - *5* synonyms	67.18	85.14	73.70	85.00	87.23	92.07	70.05	79.33	58.38	82.25	28.70	53.33	62.53	74.50	73.70	89.50
+ Vector-based - *10* synonyms	**67.66** †	85.24	73.85 †	84.96	87.40	93.14	70.30	79.31	57.81	82.27	28.73	53.86	63.09	75.14	75.02	89.28
+ Vector-based - definition	67.47	83.49	72.68	84.41	86.08	91.64	67.15	78.83	59.62	81.43	29.26 †	54.77	63.73	76.40	75.69 †	91.16
+ Vector-based - standard name	63.10	83.49	70.83	84.87	88.43 †	96.14	72.05 †	84.15 †	55.00	83.10	29.26 †	54.46	65.84	80.63	74.03	93.37
+ Vector-based - *All-in-One*	67.47	82.33	**73.95**	85.33	**92.93**	96.74	**74.78**	83.85	58.15	84.57	28.79	58.52	**70.49**	81.61	**80.44**	95.02
+ Rank-based - *1* synonym	61.35	82.42	67.80	83.98	79.01	90.62	61.48	77.49	56.67	80.03	26.72	51.70	53.02	71.40	66.85	85.19
+ Rank-based - *3* synonyms	62.03	85.24	69.07	84.53	81.28	94.38	64.44	80.16	57.21	83.55	27.73	54.58	55.56	73.87	67.84	86.29
+ Rank-based - *5* synonyms	63.68	85.43	69.69	84.72	82.18	94.47	64.52	80.18	58.74	84.53	27.79	54.80	56.83	74.29	69.06	88.06
+ Rank-based - *10* synonyms	63.59	85.92	70.46	84.81	81.92	94.98	64.79	81.10	58.86	85.28	27.66	56.49 †	57.67	75.21	70.27	89.06
+ Rank-based - definition	66.01	84.46	71.45	85.33	83.51	94.64	65.69	79.14	57.66	82.41	26.91	56.02	58.45	77.46	69.06	90.60
+ Rank-based - standard name	62.13	84.95 †	70.52	**85.80** †	83.08	96.78 †	69.34	83.83	62.27 †	85.95 †	29.26 †	55.71	59.15	80.98 †	69.06	94.47 †
+ Rank-based - *All-in-One*	65.72	**86.40**	73.61	85.67	92.84	**96.83**	74.05	**84.77**	**66.50**	**89.41**	**30.45**	**59.62**	68.66	**81.76**	78.89	**95.13**

Table 5. GRF performance with BioSyn (upper) and SapBERT (bottom) on refined test sets. **Bold** indicates best and underlined second-best results overall. † indicates the best standalone generative feedback type. Values below the baseline are colored in red.

R. Islamaj, C.-H. Wei, D. Cissel, N. Miliaras, O. Printseva, O. Rodionov, K. Sekiya, J. Ward, and Z. Lu. Nlm-gene, a richly annotated gold standard dataset for gene entities that addresses ambiguity and multi-species gene recognition. *Journal of biomedical informatics*, 118:103779, 2021. ISSN 1532-0480. doi: 10.1016/j.jbi.2021.103779.

R. Islamaj, R. Leaman, D. Cissel, C. Coss, J. Denicola, C. Fisher, R. Guzman, P. G. Kochar, N. Miliaras, Z. Punske, K. Sekiya, D. Trinh, D. Whitman, S. Schmidt, and Z. Lu. Nlm-chem-bc7: manually annotated full-text resources for chemical entity annotation and indexing in biomedical articles. *Database*, 2022, 12 2022. doi: 10.1093/database/baac102.

J. Lee, W. Yoon, S. Kim, D. Kim, S. Kim, C. H. So, and J. Kang. Biobert: a pre-trained biomedical language representation model for biomedical text mining. *Bioinformatics*, 36:1234 – 1240, 2019. URL https://api.semanticscholar.org/CorpusID:59291975.

J. Li, Y. Sun, R. J. Johnson, D. Sciaky, C.-H. Wei, R. Leaman, A. P. Davis, C. J. Mattingly, T. C. Wiegers, and Z. Lu. Biocreative v cdr task corpus: a resource for chemical disease relation extraction. *Database*, 2016(baw068), 2016. ISSN 1758-0463. doi: 10.1093/database/baw068.

C. E. Lipscomb. Medical subject headings (mesh). *Bulletin of the Medical Library Association*, 88(3):265, 2000.

L. Luo, C.-H. Wei, P.-T. Lai, Q. Chen, R. I. Dogan, and Z. Lu. Assigning species information to corresponding genes by a sequence labeling framework. *Database : the journal of biological databases and curation*, 2022, 2022. URL https://api.semanticscholar.org/CorpusID:248572309.

G. Martin, N. Goran, and B. Casey M. Linnaeus: A species name identification system for biomedical literature. *BMC Bioinformatics*, 11, 2010. ISSN 1471-2105. doi: 10.1186/1471-2105-11-85.

A. A. Morgan, Z. Lu, X. Wang, A. M. Cohen, J. Fluck, P. Ruch, A. Divoli, K. Fundel, R. Leaman, J. Hakenberg, C. Sun, H.-h. Liu, R. Torres, M. Krauthammer, W. W. Lau, H. Liu, C.-N. Hsu, M. Schuemie, K. B. Cohen, and L. Hirschman. Overview of biocreative

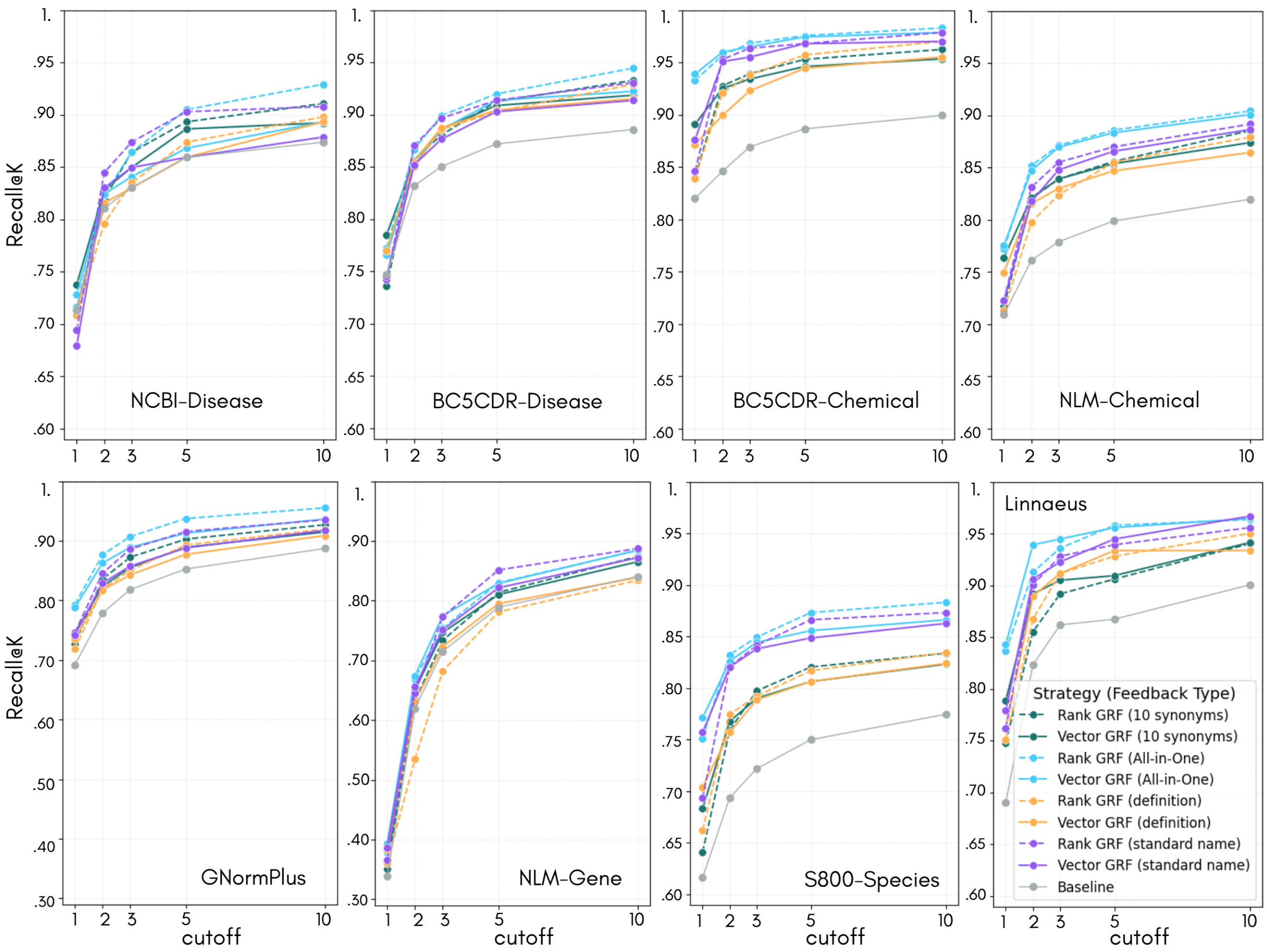

Fig. 2: Recall curves for BioSyn, showing recall@k for different generative feedback types and integration strategies (vector-based GRF is indicated with solid lines, rank-based GRF with dashed lines), compared to the GRF-free baseline (solid grey line).

ii gene normalization. *Genome Biology*, 9:S3, 2008. doi: 10.1186/gb-2008-9-s2-s3.

E. Pafilis, S. P. Frankild, L. Fanini, S. Faulwetter, C. Pavloudi, A. Vasileiadou, C. Arvanitidis, and L. J. Jensen. The species and organisms resources for fast and accurate identification of taxonomic names in text. *PLOS ONE*, 8(6):e65390, 2013. ISSN 1932-6203. doi: 10.1371/journal.pone.0065390.

F. Scott. The ncbi taxonomy database. *Nucleic Acids Research*, 40:D136–D143, 1 2012. ISSN 0305-1048. doi: 10.1093/nar/gkr1178.

C.-H. Wei, H.-Y. Kao, and Z. Lu. Gnormplus: An integrative approach for tagging genes, gene families, and protein domains. *BioMed Research International*, 2015:e918710, 2015. ISSN 2314-6133. doi: 10.1155/2015/918710.

S. Zhang, H. Cheng, S. Vashishth, C. Wong, J. Xiao, X. Liu, T. Naumann, J. Gao, and H. Poon. Knowledge-rich self-supervision for biomedical entity linking. In *Findings of the Association for Computational Linguistics: EMNLP 2022*, pages 868–880. Association for Computational Linguistics, 2022.

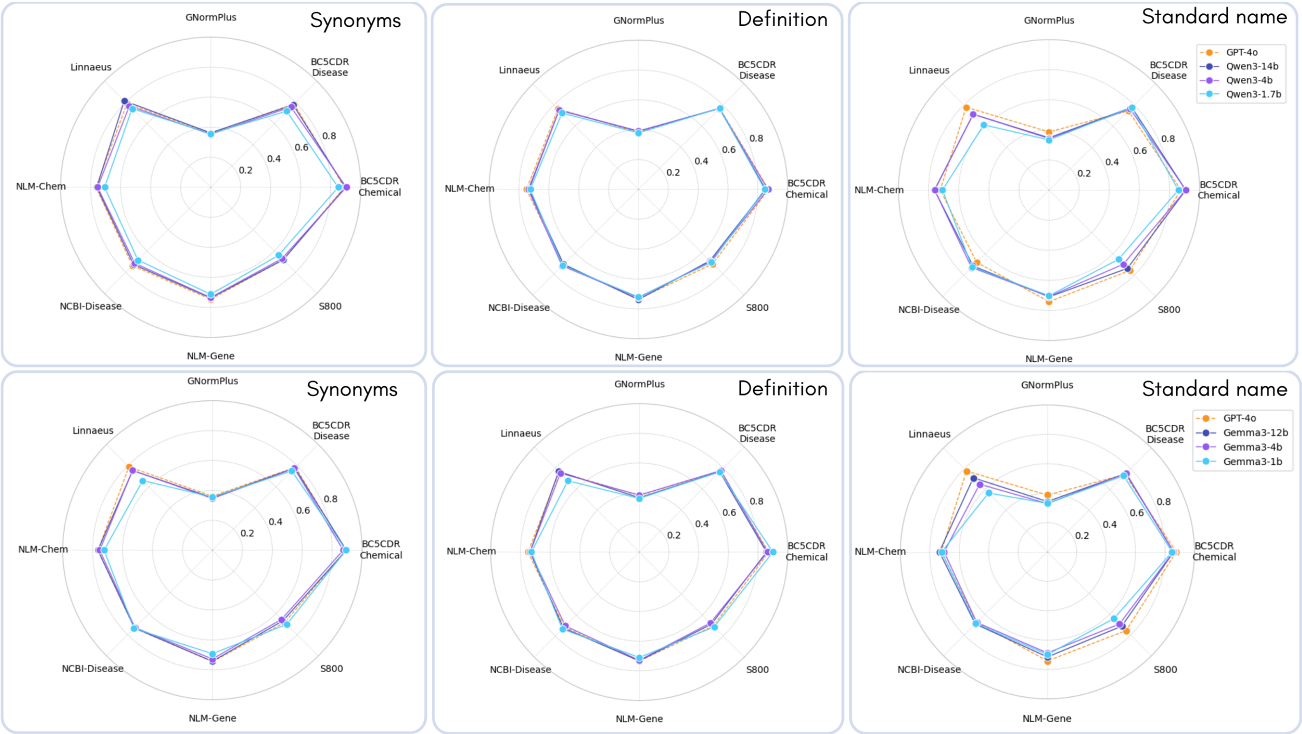

Fig. 3: Comparison of retrieval accuracy using feedback generated by the `Qwen-3` (upper row) and `Gemma-3` (bottom row) small open-source model families, with `GPT-4o` feedback shown as a gold dashed reference line. Within each model family, a graded blue color scheme indicates model size, ranging from dark blue (for models with >10B parameters) to light blue (for models with 1B parameters). Retrieval performance is evaluated using fine-tuned BioSyn under the vector-based GRF using *Synonyms* (left), *Definition* (center) and *Standard name* (right) feedback categories.

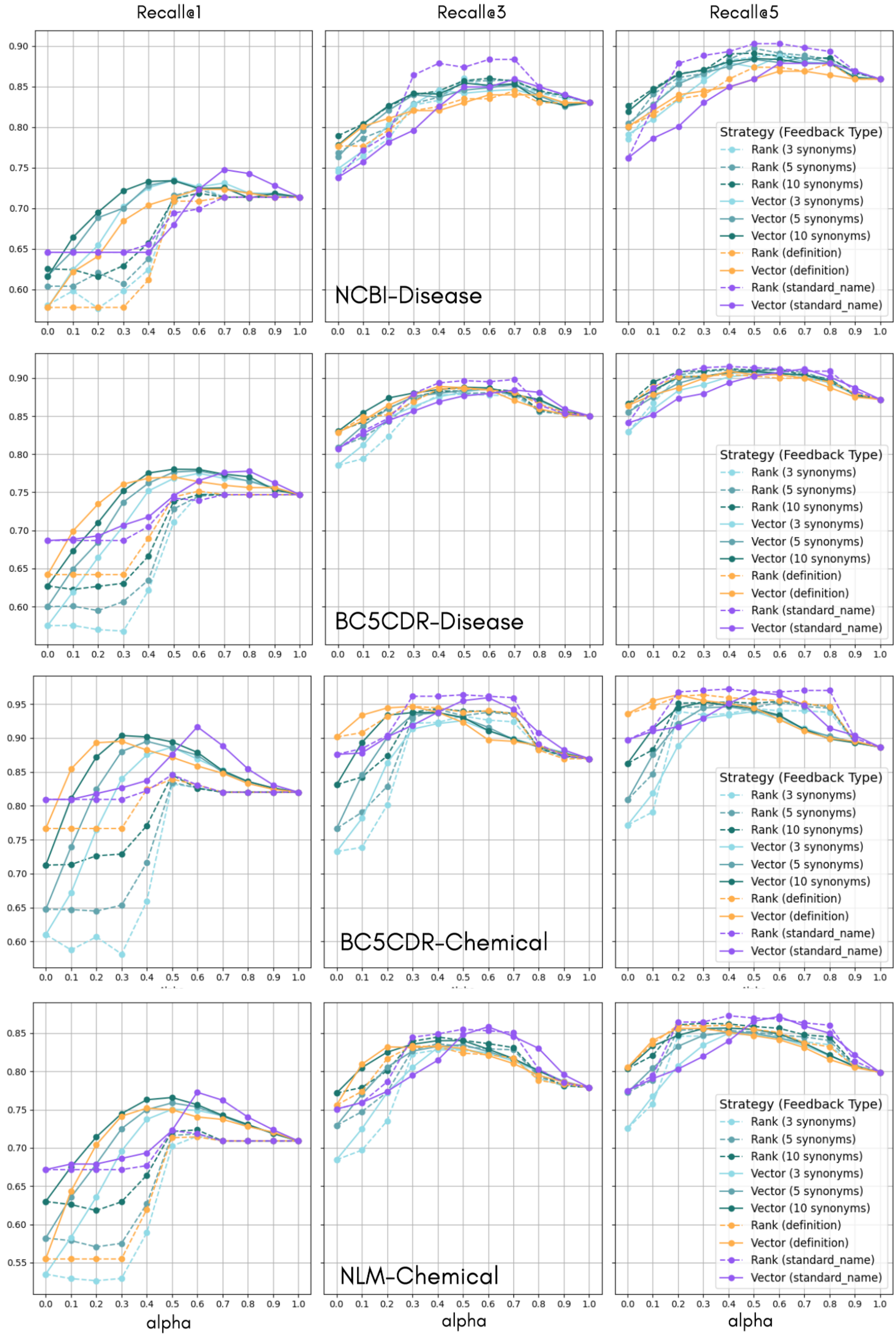

Fig. 4: Recall@k ($k \in \{1, 3, 5\}$) for different generative feedback types and integration strategies across varying values of α.

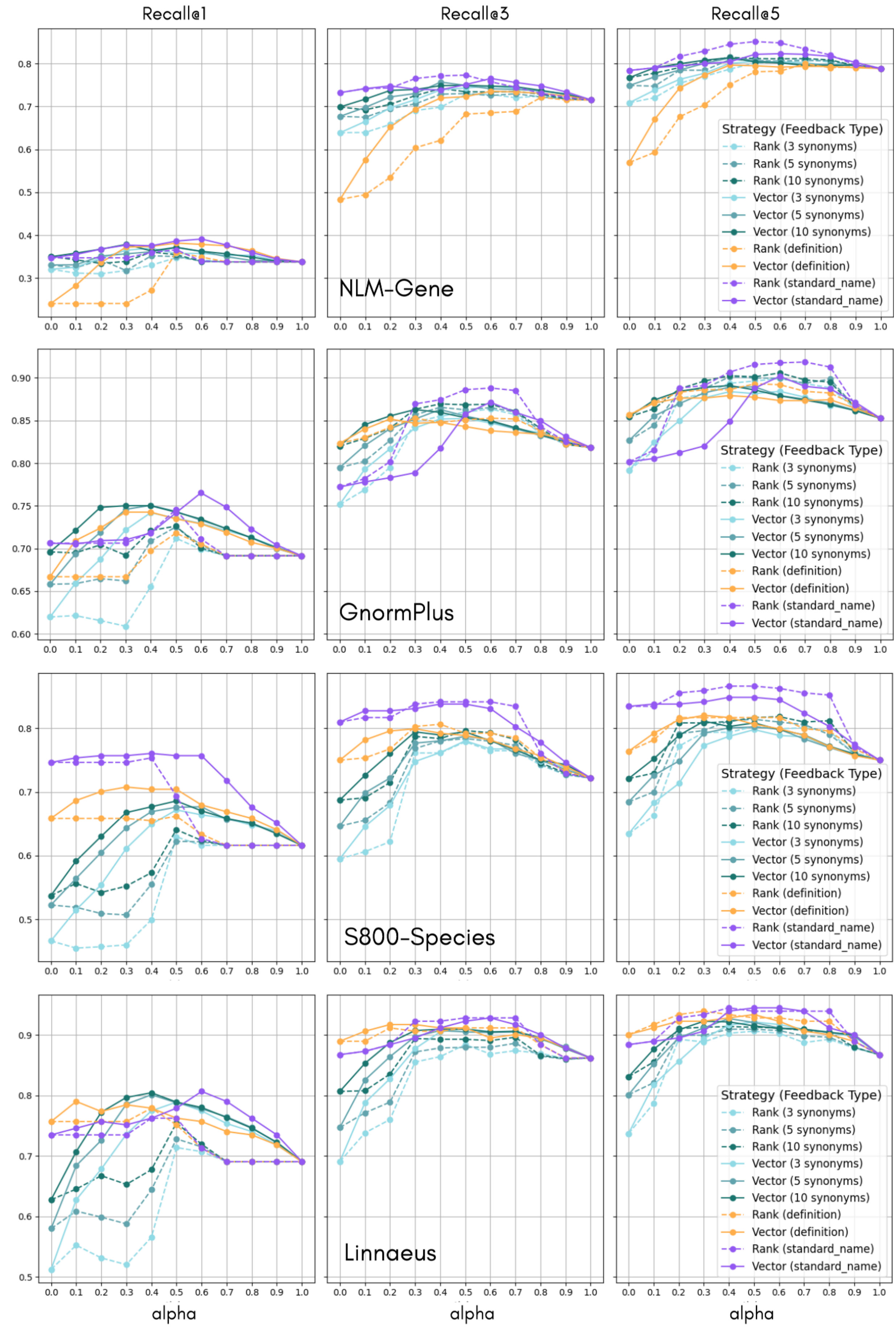

Fig. 5: Recall@k ($k \in \{1, 3, 5\}$) for different generative feedback types and integration strategies across varying values of α.

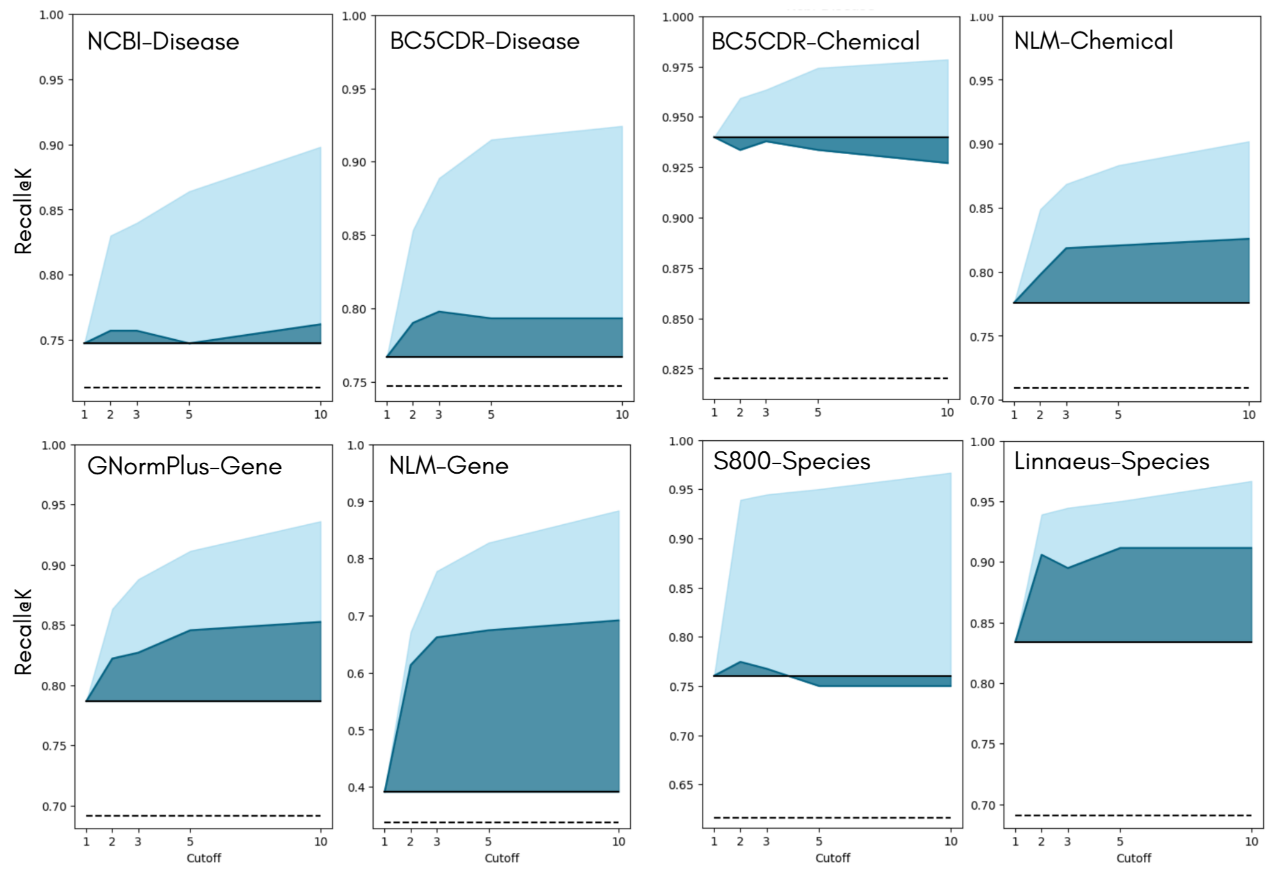

Fig. 6: Comparison of the accuracy of the RAG-based normalization method against the potential recall values at varying cutoffs. The dashed black line represents the GRF-free baseline, while the solid black line corresponds to the top-1 normalization strategy leveraging GRF-based retrieval results, which selects the first retrieved candidate as the final linking prediction. The dark blue area indicates the accuracy achieved by the RAG-based normalization method using k in-prompt candidates (where k corresponds to the cutoff), and the light blue area represents the performance gap between the re-ranking method and the potential recall across different cutoffs.
